# Society of the Red Cross

**Published:** 1882-03

**Authors:** 


### Society of the Red Cross.

The International Society of the Red Cross has issued a circular offering a prize of two thousand francs for each one of three essays on the best methods for obtaining prompt and speedy assistance for sick and wounded soldiers during a campaign. The following are the subjects proposed:

1st. The improvising of means of treatment.

2d. The improvising of means of transport.

3d. The improvising of an ambulance or field hospital.

The rules governing this competition do not differ essentially from those usually obtaining in similar trials, and are scarcely necessary to be enumerated. The subjoined are four of the most important:

1. The essays must be in manuscript and unpublished.
2. They can be written in French, or German, or English.
3. They must be forwarded to the President du Comite international de la Croix rouge, rue de l'Athenee, 8, a Geneve (Suisse), before the 1st of April, 1883.
4. Each essay shall bear a device, which must be repeated in a sealed envelope accompanying it, containing the name and address of the author.

The military character of these essays may seem to require a knowledge on many practical points beyond the attainment of those likely to enter the list for the prize; but an intelligent appreciation of the situation on the field of battle will enable a writer, even in the civil ranks of life, to furnish such ideas as ready ingenuity could suggest in an emergency; and to physicians who have served in our late war an opportunity is afforded for a public expression of valuable views drawn from that experience.

